# Spectrum of MECP2 mutations in Vietnamese patients with RETT syndrome

**DOI:** 10.1186/s12881-018-0658-x

**Published:** 2018-08-06

**Authors:** Huong Le Thi Thanh, Trinh Do Thi Diem, Chinh Vu Duy, Ha Ly Thi Thanh, Hoa Bui Thi Phuong, Liem Nguyen Thanh

**Affiliations:** 1 Department of Gene Technology - Vinmec Research Institute of Stem cell and Gene Technology, 458 Minh Khai Street, Hanoi, Hai Ba Trung district Vietnam; 2 Vinmec International Hospital, 458 Minh Khai Street, Hai Ba Trung District, Hanoi, Vietnam

**Keywords:** RETT syndrome, Neurodevelopmental disorder, MECP2, Mutation spectrum, de novo, Novel mutation

## Abstract

**Background:**

Rett syndrome (RTT) is a severe neurodevelopmental disorder in children characterized by a normal neurodevelopmental process in the first 6–18 months followed by a period of motor and vocal deterioration with stereotypic hand movements. Incidence of RTT is mostly due to de novo mutation in the MECP2 gene (methyl-CpG-binding protein 2).

**Methods:**

The study assessed 27 female patients presented with classical RTT phenotype age range from 18 months to 48 months. Specialist carried out the clinical evaluation and diagnosis according to RTT diagnosis criteria. Blood samples from patients were then collected for genomic DNA extraction. We next performed MECP2 gene amplification and sequencing of the whole coding region to screen for mutations.

**Result:**

MECP2 mutation was found in 20 patients (74%) including: 2 missense, 4 nonsense, 6 frameshift and 2 deletion mutation. The study identified 14 pathogenic mutations which we found 4 mutation, to our knowledge and extensive search, not priory reported in any mutation database or publication: c.1384-1385DelGT, c.1205insT, c.717delC and c.1132_1207del77. High percentage of C > T (70%) in CpG sites mutation was found.

**Conclusion:**

Our result reveals a high percentage of C > T mutation in CpG hot spot, which is more prone to modification and more likely to be detected in RTT as a disorder is strictly due to de novo mutations. The study is the first to identify the mutation spectrum of MECP2 gene in Vietnamese patients and also an important step toward better diagnosis and care for RTT patients in Vietnam.

## Background

Rett syndrome (RTT; OMIM Entry #312750) is a neurodevelopmental disorder which is one of the main causes of neurological disability in children [1]. Rett syndrome almost occurs in females, the estimate incident of RTT worldwide is 1:10.000–1:15000 female births [2]. The classic clinical presentation of RTT is characterized by average psychomotor progression from birth until 6–18 months, and then development halted followed by period of psychomotor deterioration [3, 4] . During the phase of deterioration, the patients often present stereotypic movements (classic RTT hand), loss of acquired motor skills and communication skills [5]. The neurodevelopmental of the child may stabilize or in some case, limited recovery can be observed in the next phase called the pseudo-stationary stage [6]. Other complications and symptoms of RTT may include seizure, cardiac abnormalities, irregular breathing patterns and scoliosis [7–9]. The level of motor impairment becomes more severe as the child’s age [10]. There are also atypical RTT patients whom present with several but not all clinical features and progression phases of classical RTT [11].

The most common cause of RTT is de novo mutations in the MECP2 gene (Methyl-CpG binding protein 2), located on the X chromosome (locus Xq28) [1]. Additionally, the gene is also subjected to X-chromosome inactivation, which alters the severity and manifestation of RTT [12]. The MECP2 gene consists of four exons and encode for the widely expressed methyl-CpG-binding protein 2 – member of the methyl-binding domain protein family. MECP2 is an important transcription factor, which functions by interaction and modification of epigenetic factors [13]. There exist two isoforms of MECP2 (MeCP2_e1 and MeCP2_e2) which have slight difference in their expression levels between tissues and distinct N termini. MeCP2_e1 is most commonly found in the brain tissue whilst MeCP2_e2 predominate in the placenta, liver and skeletal muscle tissues [14, 15]. Many studies have addressed MECP2 role in RTT, beside the critical role in neurodevelopmental process, MECP2 have been hypothesized to help maintenance of synaptic function, brain cell connectivity and neuronal plasticity [16, 17].

To date, around 1000 disease-causing MECP2 mutations have been characterized and recorded in multiple databases such as RettBASE and The Human Gene Mutation Database [18]. Case reports of asymptomatic carriers or carriers with mild clinical symptoms have been found to have skewed X chromosome inactivation (XCI) in preference of the normal copy of MECP2 [12]. In addition, MECP2 mutations have been loosely associated with other pediatric neurological conditions, most notably autism, Angelman syndrome, and many behavioral and intellectual disorders [19, 20].

In Vietnam, the lack of awareness and knowledge is also a major problem as consultation measures need to be provided families of RTT patients. Diagnosis of RTT require both clinical and molecular evidence, in which characterization of MECP2 gene is required for the disorder and provide valuable information for clinician in planning care and treatments.

In this study we first report the mutation spectrum of MECP2 in Vietnamese patients with clinically diagnosed for Rett syndrome, in which 4 novel mutations was found. This finding is an important step toward understanding and development of therapy to help patients suffering from RTT.

## Methods

### Study population

The study assessed 27 female patients presented with sporadic, RTT phenotype; diagnosis were made based on the criteria by Neul et al. [11]. Specialized pediatricians and neurologists at Vinmec hospital made clinical evaluation and confirmation of RTT. The Ethical board of Vinmec International Hospital approved of the study; informed consent was obtained from the parents of the patients.

### Inclusion criteria

Female patients were recruited in the study based on classic presentation of RETT syndrome: average psychomotor progression from birth until 6–18 months of age, with no history of brain injury, trauma or serious infection. The patients presented with 2 out of 4 main clinical criteria:

- Loss of acquired hand skills
- Loss of acquired language skill
- Gait abnormalities: impaired (dyspraxic) or absence of ability
- Stereotypic hand movements

### Molecular analysis

The genomic DNA was extracted from patient’s peripheral blood using the Wizard® Genomic DNA Purification Kit (Promega). The DNA quality was tested by the NanoDrop spectrophotometer. In order to perform mutant analysis, we designed primers to amplify the complete coding region (exon 2, 3, 4) including flanking intronic splice site sequence and 5’UTR within exon 2 and 3’UTR based on published genomic sequence of MECP2.

DNA was amplified by manual PCR method using GoTaq® Green Master Mix (Promega) on the GeneAmp PCR system 9700 thermocycler. The PCR product was checked on agarose gel and purified using QIAquick PCR purification Kit (Qiagen) according to the manufacturer’s instruction. The purified PCR product was sequenced directly in both strands using the BigDye® Terminator v3.1 Cycle Sequencing Kit (Thermo Fisher Scientific) by automated fluorescent sequencing technology (Applied Biosystems 3500 Dx Genetic Analyzer). We used DNAstar to compare the patients’ MECP2 sequences with the reference MECP2 sequence (Genebank accession number. MECP2 locus, AF030876; MECP2, ENSG00000169057). The novel mutation was confirmed by doing extensive search on RettBASE and The Human Gene Mutation Database as well as from prior publications. We used in silico analysis to predict the consequence of the mutation identified in our cohort. The tool used were Polyphen-2 [21] and MutationTaster [22].

## Results

### Mutation analysis of the MECP2 gene

Mutation analysis was conducted in 27 female patients. The diagnosis followed the standard protocol: After clinical evaluation, blood sample was taken and PCR with the designated primers (Table 1) was used to amplify MECP2, followed by Sanger sequencing of the exons and compare with reference sequence to detect mutations. On clinical examination, all patients presented with history of a period of neurodevelopmental regression followed by recovery or stabilization, together with partial or complete loss of motor and language skill and stereotypic hand movements.

Mutation analysis detected MECP2 mutation in 20 out of 27 patients (mutation detection rate 74%)(Table 2). The study identified 14 pathogenic mutations, including 2 missenses, 4 nonsenses, 6 frame shifts and 2 deletions. The mutations that occur more frequently were c.473 C > T (*n* = 4); c.808 C > T (*n* = 4), c.763 C > T (*n* = 2) and c.502C > T (*n* = 2). All mutation were predicted to be disease causing by in silico analysis (Table 3).

**Table 1:** Primers used in MECP2 amplification

Amplicon	Length (bp)	Forward primer	Reverse primer
Exon 2	355	TAGCCCTGGGAAAAAGGTCG	GGCACAGTTTGGCACAGTTAT
Exon 3	484	CCTGCCTCTGCTCACTTGTT	CATGAGGGATCCTTGTCCCTG
Exon 4.1	429	TGCCCTATCTCTGACATTGCT	ATGACCTGGGTGGATGTGGT
Exon 4.2	631	GTCCTGGGAAGCTCCTTGTC	CTCTCCAGTGAGCCTCCTCT
Exon 4.3	388	CAGCGTCTGCAAAGAGGAGA	CCCTGAAGCCACGAAACTCT

**Table 2:** Mutation alleles frequency of RETT patients

Patient No.	Mutation of *MECP2*		Mutation type	Novel mutation
	Nucleotide change	Amino acid change		
1–4	c.473C > T	p.T158 M	Missense	No
5–8	c.808C > T	p. R270X	Nonsense	No
9–10	c.763C > T	p.R255X	Nonsense	No
11–12	c.502C > T,	p. R168X	Nonsense	No
13	c.1384-1385del GT	V462fs	Frameshift	Yes
14	c.763C > T	p.R255X	Nonsense	No
	c.1205insT	p.P402Lfs	Frameshift	Yes
15	Exon 4 deletion	_	Large deletion	No
16	c.(164–182)del 19	p.Q56fs	Frameshift	No
	(1148–1193)del 46	p.L383fs	Frameshift	No
17	c.717del C	p.A240fs	Frameshift	Yes
18	c.806del G	p. G269fs	Frameshift	No
19	c.1132_1207del77	A378fs	Deletion	Yes
20	c.917G > A	p.R306H	Missense	No

**Table 3:** In silico analysis to determine the causal relationship of the mutation and RETT syndrome

Nucleotide change	Amino acid change	Domain	MutationTaster	PolyPhen-2	Reference
c.473C > T	p.T158 M	MDB	Disease causing	Probably damaging with a score of 1.000	Rettbase
c.808C > T	p. R270X	TRD-NLS	Disease causing	N/A	Rettbase
c.763C > T	p.R255X	TRD	Disease causing	N/A	Rettbase
c.502C > T,	p. R168X	Inter-domain region	Disease causing	N/A	Rettbase
c.1384-1385del GT	p.V462fs	C- term	Disease causing	N/A	Novel
c.763C > T	p.R255X	TRD	Disease causing	N/A	Rettbase
c.1205insT	p.402Lfs	C - term	Disease causing	N/A	Novel
c.(164–182)del 19	p.Q56fs	TRD-NLS, 3’UTR	Disease causing	N/A	Rettbase
c.(1148–1193)del 46	p.L383fs	C - term	Disease causing	N/A	Rettbase
c.717delC	p.A240fs	TRD	Disease causing	N/A	Novel
c.806delG	p. G269fs	TRD-NLS	Disease causing	N/A	Rettbase
c.1132_1207del77	A378fs	C – term	Disease causing	N/A	Novel
c.917G > A	p.R306H	TRD	Disease causing	Probably damaging with a score of 0.999	Rettbase

N/A: Not applicable

**Fig. 1:**
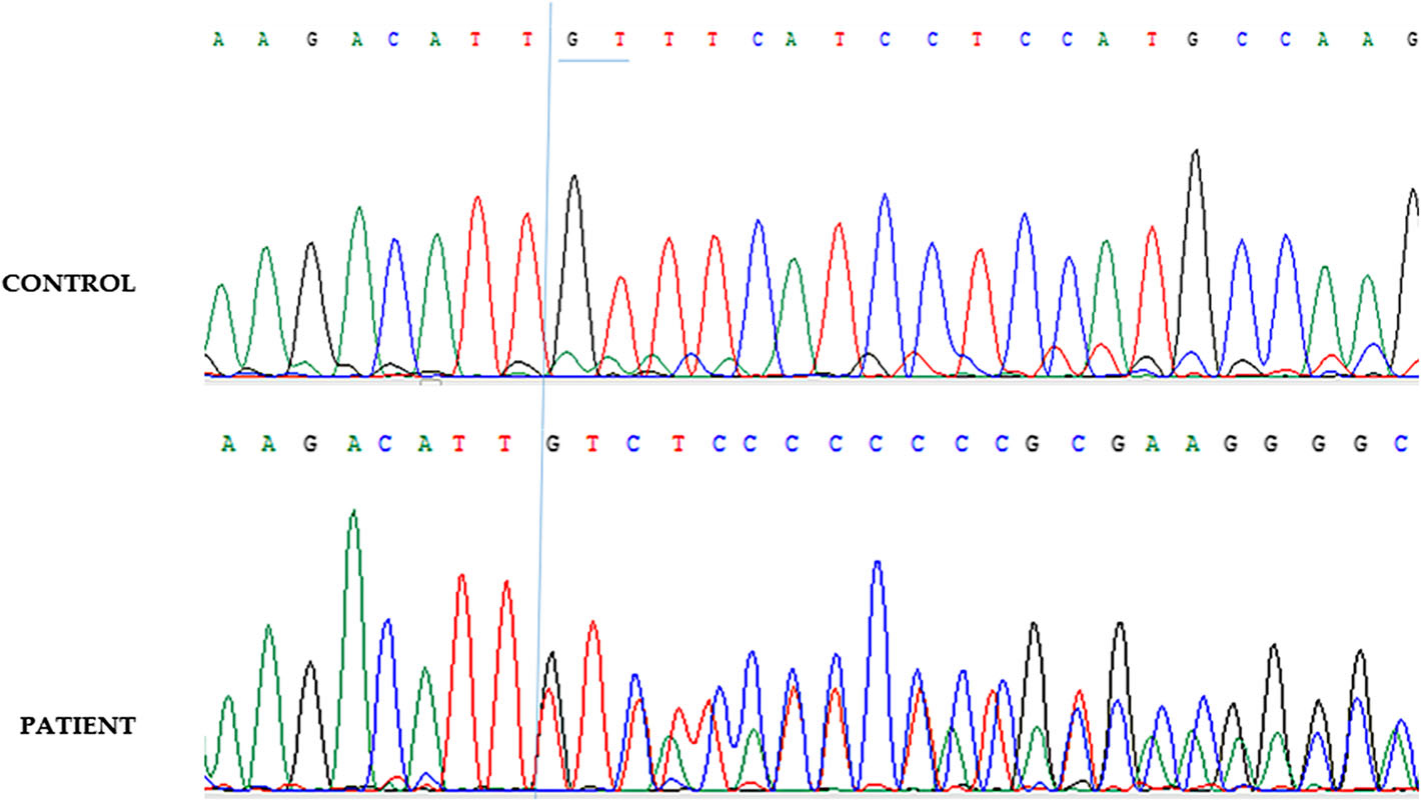
Sanger sequencing result of the patient with novel mutation (c.1384–1385 del GT)

A novel mutation was also identified: c.1384–1385 del GT (Fig. 1), a two-nucleotide deletion which lead to frameshift and alter the amino-acid sequence and premature termination of the protein product. The carrier was a female patient diagnosed with classic RTT at the age of 3. We also identified 3 other novel mutations: c.1205insT, c.717delC and c.1132_1207del77, to the best of our knowledge, not described in the MECP2 mutation database (Rettbase) or previously described in any publication.

### In silico prediction of mutation

Two in silico tool (Polyphen-2, MutationTaster) were used to predict the consequence of mutation. We identified the region change, probability of disease causing and potential changes to protein structure of each mutation identified in our cohort.

## Discussion

The current study investigated the mutation spectrum of MECP2 in Vietnamese patients. The patients had been enrolled and pediatricians and neurologists carried out clinical evaluation. Therefore, the cohort is well defined and well suited for molecular study. Prior to availability of MECP2 mutation test, criteria for Rett syndrome diagnosis are based on a collection of clinical features organized into age-related stages [23]. Classic Rett syndrome was classified for girls meeting all main criteria while variants of this disorder were marked for patients with a less severe clinical presentation, including preserved speech or hand use, normal head circumference, or delayed symptom onset.

Mutations of MECP2 gene are highly correlated with RTT and have been found in other neurological disorder such as autism, Angelman syndrome, and other behavioral and intellectual disorders. Therefor molecular diagnosis for MECP2 mutations is an important step in diagnosis of neurodevelopmental disorders. Routine diagnostic protocol for MECP2 mutations is often carried out by DNA sequencing because of the gene’s broad spectrum of mutation.

We identified MECP2 mutation in 74% of the cases, the mutation detection rate is comparable with other study in which MECP2 mutations were identified in approximately 75% of RTT patients [24–26] . The result suggest that MECP2 mutation is not the only cause of RTT, as there are also FOXG1 gene (locus 14q13) which causes the congenital variant of Rett syndrome and others mutations which could also lead to RTT [27]. Furthermore, we did not take into account intronic sequence change (i.e. splice site mutation) which could lead to changes in the protein structure and function. Therefore, the absent of MECP2 alone is not enough to rule out the possibility of RTT in cases where the child does not have typical symptoms or not old enough to conclude with the diagnosis of classic RTT.

The study identified 14 pathogenic mutations, including 2 missenses, 4 nonsenses, 6 frame shifts and 2 deletions. The more frequently detected mutations in our cohort (c.473 C > T; c.808 C > T, c.763 C > T and c.502C > T) are also the most common mutations reported worldwide as listed on RettBASE [18]. The mutation profile suggested that the similarity of the mutation spectrum in MECP2 is not the product of heredity, but the susceptibility of the mutation sites. We noted that most of the point mutations in our cohort are C > T in CpG sites. The CpG sites are often mutation hotspot because the cytosine in these sites is often methylated (mCpG) and 5-methylcytosine is genetically unstable [28]. In disorder like RTT, which is strictly due to de novo mutations, these CpG sites mutations became more frequent.

In our cohort, we detected a novel mutation not listed previously in any mutation databases: c.1384-1385delGT. The patient had clear clinical presentations of classic RTT. The patients had a relatively normal neurodevelopmental phase in the first 8 months, and then the child’s development regressed. At 12 months of age, she experienced a seizure and was taken to the hospital. At 3 years of age, she was unable to walk independently, and stereotypic hand movements hindered hand usage. She exhibited poor interaction to her surrounding and to other people, and show no communication ability. In silico analysis confirmed the mutation as a disease causing variant.

Before the availability of MECP2 mutation test in Vietnam, criteria for the diagnosis of Rett were solely based on clinical findings. This could lead to the under-diagnosis of Rett thus, overlooking the milder form of the disease. In order to better manage and reduce the incident of Rett we need to develop a framework to provide counseling, prenatal diagnosis for the patients and families.

## Conclusions

In conclusion, the study has identified and characterized a spectrum of MECP2 mutations in sporadic case of Rett syndrome and patients with Rett like feature. Because of the clinical consequence and also the lack of data of Rett syndrome in Vietnam, future studies need to be conducted on a larger scale in different parts of the country and could provide better counseling for the patients as well as their families. The mutation spectrum of Vietnamese patients will also contribute to the global mutations database and help progress toward better understanding the etiology of the disorder and aid treatment in the near future.

## Abbreviations

MECP2: Methyl CpG binding protein 2
MLPA: Multiplex Ligation-dependent Probe Amplification
PCR: Polymerase Chain Reaction
RTT: Rett Syndrome
